# Efficacy of lotilaner (Credelio™) against experimentally induced infestations of the adult cat flea, *Ctenocephalides felis*, and flea eggs following oral administration to cats

**DOI:** 10.1186/s13071-021-04660-2

**Published:** 2021-03-05

**Authors:** Tandy Paarlberg, Daniela Karadzovska, Rainer Helbig

**Affiliations:** 1 Elanco Animal Health, 2500 Innovation Way, Greenfield, IN 46140 USA; 2 Elanco Australasia Pty Ltd, 245 Western Road, Kemps Creek, NSW 2178 Australia; 3 Elanco Animal Health, Mattenstrasse 24a, 4058 Basel, Switzerland

**Keywords:** Lotilaner, Fleas, Cat, Efficacy, Flea egg production

## Abstract

**Background:**

Credelio™ (lotilaner; Elanco) is indicated for the treatment of flea and tick infestations on cats at a recommended lotilaner dose rate of 6–24 mg/kg. This study evaluated the efficacy and safety of lotilaner following a single oral administration to cats for the treatment and prevention of adult *Ctenocephalides felis* fleas and flea egg production under laboratory conditions.

**Methods:**

Two treatment groups of ten cats each were used in this study. One group was treated with lotilaner at a dose rate of 6−9 mg/kg on Day 0 and the other group served as the control group. Each cat was infested with 100 unfed adult fleas on days –1, 6, 13, 20 and 29. At 24 h post-treatment or post-infestation, each cat was combed to remove and count adult live fleas. At each time point, flea eggs were also collected and counted from under each cat cage.

**Results:**

Following a single oral administration of lotilaner at a minimum dose rate of 6 mg/kg (range 6.00−8.57 mg/kg), the lotilaner group displayed 100%, 100%, 99.9%, 99.9% and 99.8% efficacy against adult live flea counts as compared to the control group on Days 1, 7, 14, 21 and 30, respectively. At each time point, adult flea counts from the lotilaner-treated cats were significantly lower (*P* < 0.0001) than from the control group. A mean flea egg count of 22.6 in the lotilaner-treated cats (compared to 441.7 in the control animals) was observed 24 h post-treatment. No eggs were present from any of the treated cats on Days 7, 14 and 30 and a single egg was detected on a single treated cat on Day 21. One adverse event (regurgitated food) was observed during the study in one treated cat approximately 1 h after dosing.

**Conclusions:**

Lotilaner was well tolerated; only one adverse event was observed in the treated group. Virtually all adult fleas were killed within 24 h post-treatment or post-infestation in cats treated with a single dose of lotilaner as compared to the control group, thus significantly reducing the number of flea eggs being produced for 30 days after treatment. 
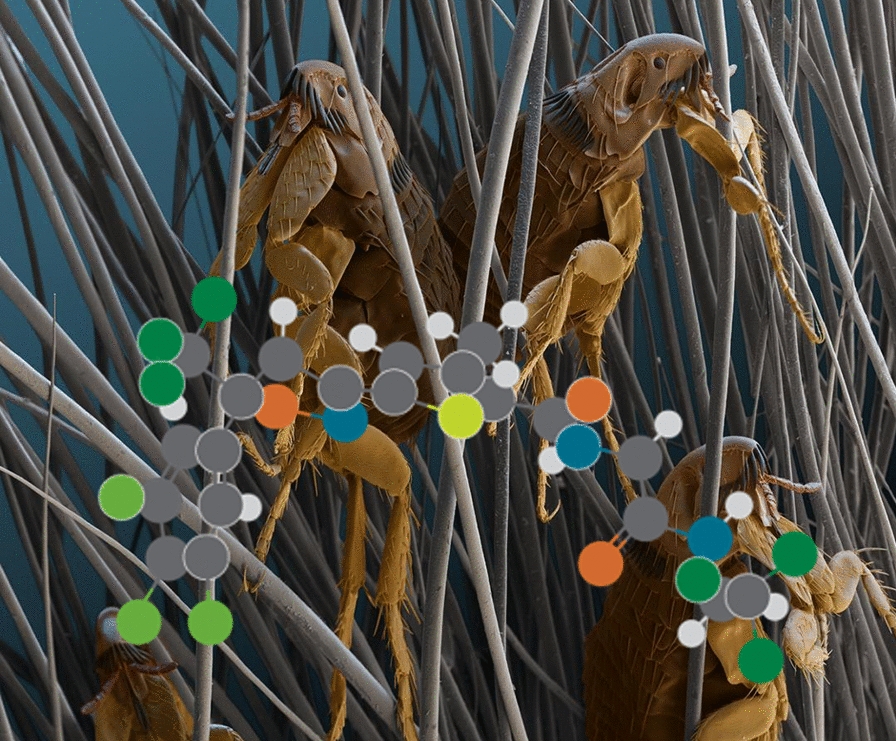

## Background

The cat flea (*Ctenocephalides felis*) is an extremely common parasite infesting the domestic cat. It is considered an important vector and contributing factor in diseases affecting cats (flea allergy dermatitis, *Dipylidium caninum* infestations) and humans (flea bite allergy) [1, 2].

Cat flea infestations in the home environment typically begin when pets carry fleas indoors. Under favorable conditions, a female cat flea can produce an average of 20–30 eggs per day during a lifespan of 3–4 weeks [3]. As cat fleas rarely leave their host voluntarily, on-host flea control becomes an important element of treatment; another is prevention of flea reproduction. The female cat fleas begin laying their eggs within 24 to 36 h of acquiring a host and feeding [2]; therefore, products providing a rapid speed of kill that eliminate female fleas prior to production of eggs are highly desirable.

There are numerous safe and effective host-targeted flea control agents available for cats as either topical (dinotefuran, fipronil, imidacloprid, indoxacarb, selamectin, spinetoram) or oral (spinosad, nitenpyram) applications [4], some of which are also effective against ticks. Most recently, a new class of parasiticides, the isoxazolines, has been introduced to the market with both topical and oral formulations. Lotilaner (Credelio™; Elanco) is the first of the isoxazolines to be registered as an oral product for cats that treats flea and tick infestations [5]. Other isoxazolines such as fluralaner (Bravecto® spot-on solution MSD/Merck), and sarolaner in combination with selamectin (Stronghold® Plus/Revolution® Plus; Zoetis) have been approved as topical spot-on applications.

The safety and efficacy of lotilaner flavored chewable tablets for cats (Credelio™, Elanco) against fleas (*C. felis*) and ticks (*Ixodes ricinus*) for 1 month, following oral administration at the minimum dose rate of 6.0 mg/kg, have been demonstrated in multiple pivotal laboratory studies [6, 7]. Safety was further demonstrated in a pivotal target animal safety study in 8-week-old kittens that showed lotilaner tablets to be safe at doses up to 130 mg lotilaner/kg of body weight when administered every 4 weeks for 8 months [8].

In this study, the efficacy against adult fleas but also egg production and the safety of lotilaner administered once, at the minimum dose rate (6.0 mg/kg body weight), to cats experimentally infested with adult fleas under laboratory conditions was evaluated.

## Methods

This study was a blinded, randomized, negative controlled laboratory study. Blinding of the study was achieved through the separation of functions; personnel conducting observations, performing flea infestations and counts were blinded to treatment allocation.

### Animals

Twenty purpose-bred cats aged between 10 and 149 months were used in this study. Cats were in good health at the start of the acclimation phase, weighed ≥ 2.7 kg and had not been treated with an ectoparasiticide for a least 8 weeks prior to the start of the study (6 months prior for isoxazoline compounds). Cats wore an Elizabethan collar (to avoid self-grooming and resulting flea removal) during each of the infestation periods and were housed in individual cages for the duration of the study. Within each cage, cats were maintained on a raised floor, so eggs and debris dropped directly onto butcher paper placed on the stainless steel cage floors at the beginning of each collection period. Cats were observed for general health at least once daily throughout the study and were fed an appropriate maintenance ration of a commercial cat food. On the day of dosing, commercial canned food was used to encourage food consumption. Water was available ad libitum.

### Animal selection and randomization

On Day –7, all cats were infested with 100 unfed adult fleas (*C. felis*; approximately 50% male and 50% female) of a laboratory bred colony (USA strain). On Day –6, flea comb counts were performed to assess the susceptibility of each cat to maintaining experimental infestations and for random allocation of cats to treatment groups. Flea combing using a fine-toothed comb was completed in accordance to the laboratory’s standard operating procedure. The 20 cats that met the inclusion criteria, none of the exclusion criteria and had the highest pre-treatment live adult flea counts were used in this study. To ensure the treatment groups were balanced with respect to flea retention, pre-treatment live adult *C. felis* infestation levels were used as the blocking factor. Cats were ranked in order of live flea count from highest to lowest. Within each block, each treatment was represented once and randomly rearranged before cats were assigned. There were ten blocks of two cats defined.

### Treatments

On the day of treatment, each cat was offered approximately 1/3 of the manufacturer’s recommended daily amount of food approximately 30 min prior to treatment. Two cats did not consume one-third of their daily ration and were treated with lotilaner.

Single or multiple combinations of lotilaner tablet sizes were used to achieve a lotilaner dose rate of 6−9 mg/kg corresponding to the lower end of the registered dose rate of 6–24 mg/kg on Day 0 post-feeding. Administration of the tablets was immediately followed by a check of each cat’s mouth to ensure the full dose had been swallowed. A standard dose of tap water (approximately 5 ml) was given via syringe after the administration of each tablet to facilitate swallowing.

Each cat in the control group was “mock dosed” on Day 0. Mock dosing involved opening the cat’s mouth and administering a standard volume of tap water (approximately 5 ml) with a syringe. Mock dosing facilitated calculating times for clinical observations post-treatment and for adult flea and flea egg counts on Day 1.

Cats were observed following treatment for any adverse events. Following the 1-h post-dosing observations, each cat was offered their remaining portion of their standard daily maintenance diet.

### Efficacy evaluations

On Days –1, 6, 13, 20 and 29 each cat was infested with approximately 100 unfed adult fleas (*C. felis*) of a laboratory bred colony (USA strain from Young Veterinary Research Services). All cats were infested as per the laboratory’s standard operating procedure.

Cats were maintained on a raised floor, so eggs and debris dropped directly onto butcher paper covering the stainless steel cage floor. On Day 0, before cats were removed from cages to be dosed, their hair coat was roughed to help remove eggs that had already been laid. During dosing of cats, the floor for each animal was swept to remove and dispose of existing eggs and debris.

Flea egg collections and adult flea counts were performed on Days 1, 7, 14, 21 and 30 at 24 h post-treatment or post-infestation. During combing sessions for fleas, only the number of live fleas on each cat was included in the count. A flea exhibiting normal behavior or with any response to external stimuli was considered live.

Flea eggs were swept and collected from the bottom of each cat cage. Flea eggs were separated from each cat’s debris (e.g. hair, food, etc.) before counting.

**Fig. 1:**
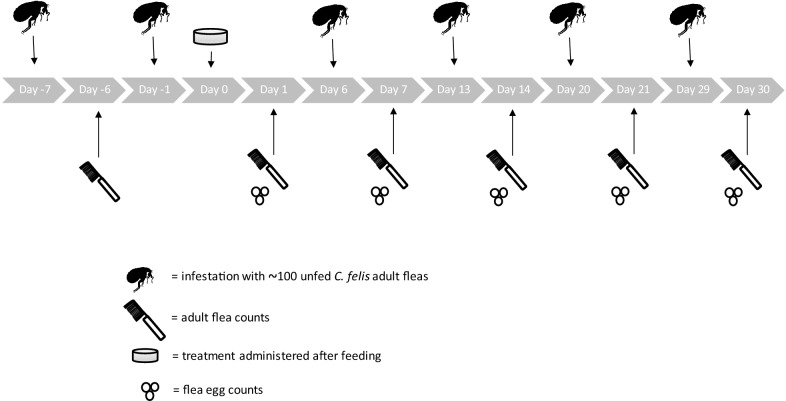
Timings of infestations, counts and treatment

The statistician prepared a list for each counting day that defined the random order (based on cage number) in which both adult flea and flea egg counts was performed. Additionally, to minimize bias, cats were randomized to cages such that a control and treated cat may have been housed next to each other (Fig. 1).

### Data analysis

Efficacy analyses were performed twice; one analysis on the intent-to-treat population (including all randomized animals) and another analysis on the per-protocol population (excluding the two cats that did not consume one-third of their daily ration). There were no notable differences in both; therefore, only the results of the intent-to-treat population are reported here. All analyses were performed using SAS/STAT® software (version 9.2, SAS Institute Inc., Cary, NC, USA).

Percent efficacy of lotilaner with respect to the untreated control group was calculated using the formula:${1}00*\left( {\left( {{\text{C}} - {\text{T}}} \right)/{\text{C}}} \right)$ where *C* arithmetic mean number of adult live fleas in the control group and *T* arithmetic mean number of adult live fleas in the lotilaner-treated group for each time point.

Comparisons of live flea counts between treatment groups were tested using the two-sided 5% significance level. A mixed model analysis was used to analyze the logarithm-transformed counts + 1 by time point, with treatment group as a fixed effect and block as a random effect. In addition, a non-parametric comparison between treatment groups was performed (Mann-Whitney U test). Comparisons of flea egg counts between treatment groups were also tested using the two-sided 5% significance level.

## Results

All cats included in the study demonstrated adequate pre-treatment live flea retention on day –6 with counts ranging from 65 to 97 fleas/cat in the control group and 64−94 in the lotilaner-treated group. Adequate flea infestations were obtained in all ten cats in the control group at each post-treatment evaluation time point with average (arithmetic mean) infestation rates ranging from 84.3 to 90.6% (Table 1).

**Table 1:** Adequacy of infestation of live adult fleas in placebo control cats

Treatment group	Subject ID	Live flea counts for day				
		1	7	14	21	30
Placebo	1	98	99	99	89	99
	2	87	79	88	74	65
	3	90	84	85	82	87
	4	85	87	95	88	85
	5	95	98	95	85	89
	6	94	94	87	93	91
	7	76	87	96	82	88
	8	99	89	89	88	99
	9	87	92	89	91	72
	10	86	76	83	71	90
Average infestation (%)		89.7	88.5	90.6	84.3	86.5
Range		76–99	76–99	83–99	71–93	65–99
No. adequately infested cats/total no. cats		10/10	10/10	10/10	10/10	10/10

Cats in the lotilaner treatment group received a dose in the range of 6.00–8.57 mg/kg (Table 2). One cat in the lotilaner-treated group was observed to have regurgitated food approximately 1 h after dosing. This was the only adverse event observed during the study. This cat was also one of the two cats that did not consume 1/3 of their daily ration prior to treatment.

**Table 2:** Treatment summary

Body weight range (kg)	Actual dose (mg)	*n*	Dose per body weight (mg/kg)		
			Mean	Min	Max
2.7–4.0	24	5	7.17	6.15	8.28
4.1–6.0	36	4	6.75	6.00	8.57
6.1–8.0	48	1	6.23	6.23	6.23

On Days 1, 7, 14, 21 and 30, in the intent-to-treat analysis the lotilaner group exhibited 100%, 100%, 99.9%, 99.9% and 99.8% effectiveness against fleas, respectively, (Table 3). Furthermore, the mean live flea counts of treated and untreated groups were significantly different (*P* < 0.0001), with lower counts in the lotilaner-treated group at each time point (Table 3). The individual flea counts for the two cats that did not consume 1/3 of their ration prior to treatment were zero on Days 1, 7, 14 and 21. On Day 30, one cat had zero fleas and the other had two.

**Table 3:** Summary of adult flea counts, percent efficacy and comparison results

Day	Intent-to-treat population				
	Number of cats flea free		Arithmetic mean (%efficacy)		Comparison
	Control (*N* = 10)	Lotilaner (*N* = 10)	Control (*N* = 10)	Lotilaner (*N* = 10)	
1	0	0	89.7	0.0 (100)	*t*_(9)_ = 181.07, *P* < 0.0001^**a**^ *Z* = 4.04, *P* < 0.0001^**a**^
7	1	10	88.5	0.0 (100)	*t*_(9)_ = 165.98, *P* < 0.0001^a^ *Z* = 4.04, *P* < 0.0001^a^
14	1	10	90.6	0.1 (99.9)	*t*_(9)_ = 62.04, *P* < 0.0001^a^ *Z* = 3.97, *P* < 0.0001^a^
21	1	9	84.3	0.1 (99.9)	*t*_(9)_ = 62.04, *P* < 0.0001^a^ *Z* = 3.97, *P* < 0.0001^a^
30	0	9	86.5	0.2 (99.8)	*t*_(9)_ = 37.55, *P* < 0.0001^a^ *Z* = 3.97, *P* < 0.0001^a^

Comparison results of both the parametric and non-parametric tests are presented

^a^Values significantly decreased in comparison to the control group

For the prevention of egg laying, efficacy can be concluded for all days. On Day 1, egg counts varied between 1 and 46 (mean 22.6) in the treated cats (intent-to-treat analysis; Table 4) and between 8 and 46 (mean 23.6) in the per-protocol analysis, compared to 205 to 677 (mean 441.7) in the control animals. On Days 7, 14 and 30, there were no eggs present on any of the treated cats. On Day 21, a single egg was detected on a single treated cat. The individual flea egg counts for the two cats that did not consume 1/3 of their ration prior to treatment were zero on Days 7, 14, 21 and 30. Flea eggs were noted for both cats on Day 1 (36 on one cat and 1 on the other).

**Table 4:** Summary of flea egg counts

Day	Intent-to-treat population			
	Number of cats egg free		Arithmetic mean	
	Control (*n* = 10)	Lotilaner (*n* = 10)	Control (*n* = 10)	Lotilaner (*n* = 10)
1	0	0	441.7	22.6
7	1	10	138.8	0.0
14	3	10	113.4	0.0
21	2	9	94.3	0.1
30	2	10	117.2	0.0

Throughout the study, egg counts in the control group ranged from 0 to 677, with at least six cats having counts in double digits on each counting date. Egg counts in the control group can therefore be considered adequate.

## Discussion

Without effective treatment, cats in a flea-infested environment would continuously be re-infested by new fleas from their environment. These fleas would seed the environment with eggs after feeding on their host and the flea life cycle would continue and contribute to the flea burden until stopped. Therefore, an essential element in flea control is treatment of the host, including treating all susceptible pets in a household, regularly and with a fast-acting, efficacious product to ensure flea egg production is halted. In this study, adult flea efficacy remained ≥ 99.8% 24 h after each infestation time point, demonstrating that one monthly treatment with lotilaner at the dose range of 6–9 mg/kg remains effective at eliminating repeated flea infestations for 30 days.

At 24 h post-treatment with lotilaner, adult flea efficacy was 100%. A similar result was seen at 24 h post-treatment in a speed of kill study using a mixed flea strain of Swiss and Danish origin [6]. In separate speed of kill study using the same mixed flea strain, lotilaner at 6 mg/kg was shown to be effective at killing fleas as early as 8 h post treatment (≥ 97.4%) and 100% effective within 24 h following subsequent weekly infestations of adult *C. felis* through 35 days post treatment [6]. Lotilaner’s sustained speed of kill is consistent with its pharmacokinetic profile, which shows following oral administration in fed cats, it is readily absorbed, with a half-life of > 4 weeks [9]. This same study showed that administration with food enhanced the absorption of lotilaner, providing close to 100% oral bioavailability, and reduced the inter-individual variability. For this reason, the statistical analysis was performed twice to assess the impact of the two cats from the lotilaner-treated group that did not consume one-third of their daily ration. One analysis included all randomized animals (the intent-to-treat population) and the other analysis excluded the two mentioned cats (the per-protocol population). The results (not shown here) indicate that there was no notable difference between the two analyses; hence, the deviation had no impact on the study outcome. Furthermore, the individual flea and egg counts for the two cats that did not consume 1/3 of their daily ration (one which also regurgitated food approximately 1 h after dosing) indicated lotilaner was efficacious in these animals.

Within 24 h of lotilaner administration, egg production was drastically impaired with a group mean of 22.6 eggs compared to a mean of 441.7 in the control cats. Flea eggs present on Day 1 are not considered a lack of efficacy since the cats were infested with fleas on Day –1 (thus fleas were on the cats for 48 h before egg count, which included 24 h before treatment). These most likely were already in production in the female fleas at the time of treatment as the first flea infestation was applied 24 h before dosing, giving the fleas time to feed and lay eggs [4].

There were no eggs present on any of the treated cats on Days 7, 14 and 30, and one single egg was found on one treated cat on Day 21. Although this may have been the result of the randomized housing, where control and treated cats were housed side by side, the egg counts in the treated group can be considered essentially zero at the re-infestation time points.

One adverse event was observed in this study, with one cat in the lotilaner-treated group having regurgitated food approximately 1 h after dosing. Despite this event, lotilaner was seen to be effective in this cat as live flea counts were zero on Days 1, 7, 14 and 21; two fleas were counted on Day 30. Similarly, egg counts for this cat were 36 on Day 1 and zero for all subsequent time points. As this was a single event, administration of lotilaner tablets to cats is considered well tolerated and safe. Furthermore, data from the target animal safety study, in which the product was administered orally every 4 weeks for 8 months, at doses up to 130 mg/kg lotilaner, in healthy kittens, 8 weeks old at study start, showed no clinically relevant treatment-related effects on clinical and clinical pathology parameters [8]. The safety and efficacy of lotilaner administration were further demonstrated in two field trials (EU and USA), in which an improvement or elimination of clinical signs of flea allergic dermatitis was also observed [10].

## Conclusions

Following a single oral administration of lotilaner (Credelio™) at a dose of 6–9 mg/kg to experimentally infested cats, a reduction ≥ 99.8% of adult fleas at 24 h post-treatment or post-infestation was seen for 30 days. This significant reduction impacted the egg production for the 30 days following treatment. Based on these results, lotilaner provides cats relief of fleas and, by killing fleas before they can lay eggs, depletes the flea stages in the cat’s environment.

## Data Availability

Due to commercial confidentiality of the research, data not included in the manuscript can only be made available to *bona fide* researchers, subject to a non-disclosure agreement.
